# Respiratory Syncytial Virus and Human Metapneumovirus Respiratory Hospitalizations and Outcomes in Colorado Adults ≥50 Years of Age: 2016–2023

**DOI:** 10.1093/infdis/jiaf266

**Published:** 2025-07-16

**Authors:** Eric A F Simões, Robert J Suss, Dhananjay Raje

**Affiliations:** Department of Pediatric Infectious Diseases, University of Colorado School of Medicine and Children's Hospital Colorado Aurora, Aurora, Colorado, USA; Samshoma Medical Research Inc, Denver, Colorado, USA; Department of Pediatric Infectious Diseases, University of Colorado School of Medicine and Children's Hospital Colorado Aurora, Aurora, Colorado, USA; Alpha Level Consultants, Nagpur, Maharashtra, India

**Keywords:** acute lower respiratory infection, epidemiology, RSV, HMPV, public health

## Abstract

**Background:**

Among older adults, respiratory syncytial virus (RSV) infection is a known cause of hospitalization, intensive care unit (ICU) admission, and mortality risk. The severity of the disease burden of human metapneumovirus (HMPV) among older adults is less well recognized. The objective of this study was to better understand risk factors for hospitalization with and outcomes in adults ≥50 years of age infected with HMPV and to compare these with RSV risk factors and outcomes.

**Methods:**

This was a retrospective cohort analysis of adults 50–88 years of age in 93 medical facilities in the Colorado Hospital Association database between 2016 and 2023. RSV and HMPV, other respiratory infections, and comorbidities were identified using *International Classification of Diseases, Tenth Revision* codes and grouped by increasing numbers of comorbidities. Multivariate logistic regression was performed to estimate the risk of the various predictors on ICU admission, and mortality for both HMPV and RSV infection, adjusted for sex, age, and comorbidity.

**Results:**

The highest risk for ICU admission was chronic obstructive pulmonary disease (COPD) (RSV: adjusted odds ratio [aOR], 2.24 [95% confidence interval {CI}, 1.81–2.77], *P* < .001; HMPV: aOR, 2.99 [95% CI, 2.13–4.19], *P* < .001), and those with neuromuscular disease without dementia (RSV: aOR, 2.33 [95% CI, 1.98–2.75], *P* < .001; HMPV: aOR, 2.22 [95% CI, 1.75–2.80], *P* < .001). Age significantly increased the odds of mortality among RSV-infected but not HMPV-infected patients. Neurological disorders with dementia were the highest comorbid risk factor for RSV mortality (aOR, 4.16 [95% CI, 3.01–5.77]; *P* < .001), in contrast to COPD for HMPV mortality (aOR, 12.44 [95% CI, 3.02–51.17]; *P* < .001).

**Conclusions:**

HMPV infection poses a unique disease burden with specific high-risk comorbidities among the older adult population distinct from that of RSV and warrants further study.

Respiratory syncytial virus (RSV) has been recognized as a cause of serious acute respiratory tract infections (ARIs) in older adults for some time [1, 2]. Its epidemiology, risk factors for hospitalization, and mortality are relatively well understood from many hospital-based studies and fewer population-based studies from the Americas, Europe, New Zealand, and Australia as well as several countries in Asia and Africa [3–11]. As of 2023, several RSV vaccines are licensed for use in older adults globally, and in the United States where the Centers for Disease Control and Prevention (CDC) has developed guidelines for their administration [12].

Human metapneumovirus (HMPV) has also been recognized as a cause of ARI in older adults [13], but much less is known about these infections, partly due to the virus only having been discovered a little over 20 years ago [14], and also partly because the burden of influenza and RSV appeared to be higher. Several earlier prospective studies that collected data on RSV also collected data on HMPV-related ARIs and lower respiratory tract infections (LRTIs) [15–17]. The Influenza Division of the CDC has been conducting surveillance for influenza-like illness (ILI) and severe acute respiratory infections (SARIs), but these have in the past been limited due to the inclusion of fever in the definition [18–20]. Given that fever is less common in RSV [2] and HMPV [13] ARI and LRTI, some modified definitions of SARI do not include fever, so results from these studies might show a higher burden of RSV and HMPV compared with the older studies of ILI and SARI [9]. However, there are still relatively few population-based estimates from large databases of the burden of HMPV in older adult populations.

Multiplex testing of viral nucleic acid in respiratory specimens has become more ubiquitous in Colorado hospitals in recent years, used mostly for the detection of influenza or severe acute respiratory syndrome coronavirus 2 (SARS-CoV-2) for which treatments are available and detection of these 2 viruses is often coupled or triple-plexed with RSV. The lower number of HMPV cases identified might be due to the lower number of patients being tested for HMPV. The changing of medical coding of medical encounters for billing purposes, from *International Classification of Diseases, Ninth Revision* (*ICD-9*) to *International Classification of Diseases, Tenth Revision* (*ICD-10*) (which includes direct codes for HMPV) in the United States in 2015–2016 [21, 22], and access to the Colorado Hospital Association (CHA) administrative database [23, 24] allowed us to address the relative lacuna of population-based studies of HMPV in the older adult population. As this database does not include laboratory testing data, RSV and HMPV infections were identified using coded diagnosis only.

In this study, we used data from the 93 hospitals in Colorado that contribute billing data to the CHA database, as a first step in determining the hospital-based burden of these respiratory infections in Colorado. The aim was to determine demographic and comorbid factors affecting outcomes associated with HMPV and RSV hospitalizations. These outcomes assessed the risk of intensive care unit (ICU) admission and mortality among HMPV- and RSV-infected hospitalized patients and the impact of demographic and underlying comorbidities on these outcomes. Length of hospitalization stay was assessed in a secondary analysis.

## METHODS

### Colorado Hospital Association

This was a claims database study. The study population included all emergency room and inpatient (including ICU) encounters between 2016 and 2023 obtained from the CHA database. The CHA claims database contains data on every hospital visit from all acute medical care hospitals for adults at 93 Colorado hospitals (except the Veterans Affairs system hospital) [23, 24]. Most rehabilitation, pediatric, and psychiatric hospitals in the state are also excluded from the 93 hospitals. The claims database contains de-identified demographic and hospital admission diagnostic data, duration, and place of stay (inpatient and ICU); it is structured such that inpatient (including ICU) encounters are distinct, nonoverlapping encounters. For a patient who experienced hospitalization and an ICU admission, the ICU admission is included in the hospitalization episode. ICU data are only reliably available from 2017 onward. Encounters were based on *ICD-10* codes for medically attended ARIs according to the classifications by Piedra et al and Greenbaum et al [25, 26], categorized as LRTI, ILI, and all other infections (other ARI). Furthermore, RSV-, influenza-, and HMPV-associated pneumonia, acute bronchitis, and various other specified respiratory diagnoses were identified. RSV was identified by *ICD-10* codes B97.4, J12.1, J20.5, and J21.0; HMPV by B97.81, J12.3, and J21.1; and influenza by codes J09–J11. The proportion of both RSV- and HMPV-associated hospital encounters that were also coded with an influenza diagnosis was also assessed, to determine the contribution to symptomatic illness; although RSV and HMPV may more frequently be asymptomatic in younger adults, the literature suggests it is less commonly so among older adults [27–30].

Laboratory testing was not done systematically at the hospitals and data are not included in this database; therefore, to identify potential changes in viral testing over time, hospitals were divided into the 5 Colorado demographic regions [31]. The total annual number of encounters was aggregated by hospital for both HMPV and RSV, including all inpatient (hospitalization) encounters. The proportion of encounters represented by each region per year was also assessed. Due to population and demographic variation among regions, differing trends in diagnosis over time may indicate differences in testing patterns geographically. In addition, to account for other testing biases, the total annual number and proportion of hospitalized patients with each infection was assessed by age group and by comorbidity.

To identify risk factors, the categories of high-risk conditions defined by Amand et al [32] were converted to *ICD-10* diagnostic codes and modified to differentiate between chronic conditions as underlying risk factors and acute outcomes. Acute outcomes included acute heart failure (distinct from chronic cardiac conditions), pulmonary embolism, and stroke (distinct from cerebrovascular conditions). These classifications were based on the *ICD* codes used by Hoshino et al, Wadhera et al, and Woodruff et al [33–35]. *ICD-10* codes were also identified for dementia, obesity, and severe obesity [33].

The CHA database has several outcome classifications for hospital discharge, including in-hospital mortality and being sent to hospice. We presumed that these patients died in hospice, though we had no way of confirming this.

### Statistical Methods

Data analysis was performed using SPSS version 26.0 (IBM Corporation, Armonk, New York, USA) software and R version 4.4.0 programming tool (R Foundation for Statistical Computing, Vienna, Austria). A comparison of demographic and comorbid conditions between patients hospitalized for RSV and for HMPV was done using Pearson χ^2^ test. The primary outcomes, the unadjusted and adjusted risk estimates of ICU admission and mortality after hospitalization, with 95% confidence intervals (CIs) were calculated using multiple logistic regression with age, sex, and comorbidities as independent predictors. The goodness of fit of the model was ascertained using the Hosmer-Lemeshow test. Analyses were conducted independently for RSV and HMPV groups. The risk of each outcome associated with the number of comorbidities was obtained after adjusting with age and sex, independently for each infection group. Since this was an exploratory descriptive analysis, the statistical significance associated with risk factors for ICU admission and mortality was presented without applying any rigid testing methods. The median length of hospital stay along with interquartile range was obtained for patients in each infection group and compared statistically using Mann-Whitney *U* test. Summarization of length of stay was obtained according to number of comorbidities; the between infection group comparison was performed using a Mann-Whitney *U* test, while within-group comparisons were done using the Kruskal-Wallis test.

#### Ethical Review

The study was approved by the Western Institutional Review Board.

## RESULTS

Out of a total of 975 664 inpatient and ICU records during the period 2016–2023, the study included 4619 RSV- and 2308 HMPV-infected patient encounters in Colorado hospitals (Table 1). Of these, 1412 (30.6%) and 635 (27.5%) were admitted to the ICU and 319 (6.9%) and 111 (4.8%) died with RSV and HMPV, respectively.

**Table 1.** Demographic and Comorbidity Distribution of Patients With Respiratory Syncytial Virus or Human Metapneumovirus Hospitalization

Characteristic	RSV (N = 7975)	HMPV (N = 3173)	*P* Value^a^
	Hospitalized (n = 4619)	Hospitalized (n = 2308)	
Age, y			
50–59	799 (17.3)	394 (17.1)	.813
60–64	606 (13.1)	306 (13.3)	.872
65–74	1469 (31.8)	741 (32.1)	.799
75–84	1301 (28.2)	669 (29.0)	.476
≥85	444 (9.6)	198 (8.6)	.162
Sex			
Female	2540 (55.0)	1385 (60.0)	<.001
Male	2079 (45.0)	923 (40.0)	<.001
Race/Ethnicity			
Asian	77 (1.7)	50 (2.2)	.144
Black	226 (4.9)	104 (4.5)	.476
Hispanic	195 (4.2)	130 (5.6)	.009
Native American	27 (0.6)	23 (1.0)	.056
White	3607 (78.1)	1737 (75.3)	.008
Other	487 (10.5)	264 (11.4)	.259
Comorbidities			
CCD without CAD and CVD	2780 (60.2)	1376 (59.6)	.650
CVD	72 (1.6)	43 (1.9)	.350
CAD	1005 (21.8)	427 (18.5)	.002
CAD + CVD	31 (0.7)	12 (0.5)	.450
CPD without asthma and COPD	1533 (33.2)	758 (32.8)	.773
Asthma	497 (10.8)	299 (13.0)	.007
COPD	1715 (37.1)	842 (36.5)	.599
Asthma + COPD	192 (4.2)	94 (4.1)	.869
Diabetes	1512 (32.7)	756 (32.8)	.986
Chronic renal disease	1500 (32.5)	756 (32.8)	.814
Anemia	1251 (27.1)	551 (23.9)	.004
Neurological/musculoskeletal without dementia	755 (16.3)	397 (17.2)	.367
Dementia	31 (0.7)	11 (0.5)	.326
Neurological/musculoskeletal + dementia	349 (7.6)	174 (7.5)	.980
Immunosuppressive disorders	752 (16.3)	388 (16.8)	.575
Malignancies	507 (11.0)	232 (10.1)	.240
Obesity	480 (10.4)	242 (10.5)	.905
Severe obesity	407 (8.8)	235 (10.2)	.064
Liver disease	285 (6.2)	113 (4.9)	.032
Other metabolic and immune disorders	73 (1.6)	31 (1.3)	.444

Data are presented as No. of patients (column %).

Abbreviations: CAD, coronary artery disease; CCD, chronic cardiac disease; COPD, chronic pulmonary obstructive disease; CPD, chronic pulmonary disease; CVD, cerebrovascular disease; HMPV, human metapneumovirus; RSV, respiratory syncytial virus.

^a^Obtained using Pearson χ^2^ test.

**Figure 1.**
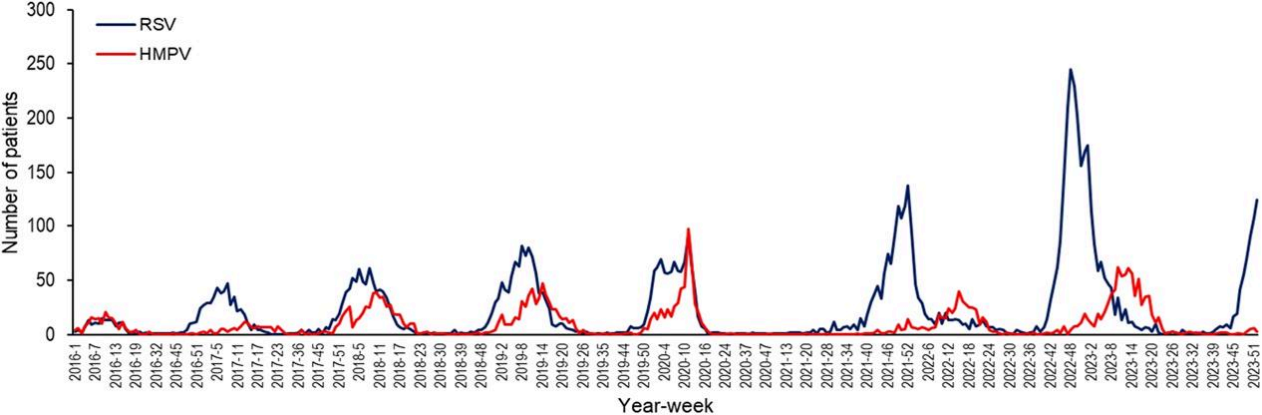
Line plot for the number of hospitalized patients with respiratory syncytial virus (RSV) and human metapneumovirus (HMPV) infections according to year and epidemiological week.

A line plot for the number of hospitalized patients with RSV and HMPV infections according to year and epidemiological week is shown in Figure 1. In the pre–coronavirus disease 2019 (COVID-19) pandemic period, the RSV and HMPV seasons roughly coincided, with the peak RSV season preceding the HMPV season by a few weeks. In the post–COVID-19 pandemic seasons of 2021–2022, 2022–2023, and early 2023–2024, the RSV season preceded the HMPV season, with the peaks not coinciding.

Case counts of RSV and HMPV hospital encounters by state region as a proportion of the total annual count are summarized in Supplementary Table 1. A total of 10 hospitals had no hospital admissions for either RSV or HMPV across all 8 seasons, including 1 pediatric hospital, 2 rehabilitation centers, 1 mental health clinic, 5 rural facilities, and 1 unidentified facility. Of the remaining 83 hospitals, 25 had <10 combined RSV- and HMPV-associated hospitalizations across all 8 years (2016–2023) and were located in the Front Range (n = 7), Eastern Plains (n = 10), or Western Slope (n = 8) regions. While the absolute number of RSV and HMPV-associated hospitalizations generally trended upward in the post–COVID-19-pandemic years (2021–2023), reporting of both infections regionally as a proportion of the annual total was overall consistent across the study period. The same trends were seen in most cases for annual hospitalizations with each infection by age group and by comorbidity; however, some comorbidities actually trended downward in postpandemic years in some cases, such as chronic obstructive pulmonary disease (COPD) without asthma, neurological conditions, and dementia (Supplementary Tables 2–4).

The proportion hospitalized with RSV- and HMPV-associated ICU admissions and deaths with influenza diagnosis by age group is shown in Supplementary Table 5. Less than 5% of hospital and ICU admissions had an influenza diagnosis across all age groups for both infections. This was generally true of the number of deaths; however, among those with both an RSV and influenza diagnosis, 2 of 23 (8.7%) of those aged 50–59 years and 7 of 55 (12.7%) of those aged ≥85 years died. As there were no deaths among those diagnosed with both HMPV and influenza (n = 115), 7 of 66 (10.6%) of those aged ≥85 years who died had a combined diagnosis of either RSV or HMPV with influenza.

### Risk Factors for ICU Admission Among Hospitalized Patients With RSV and HMPV Infections

**Figure 2.**
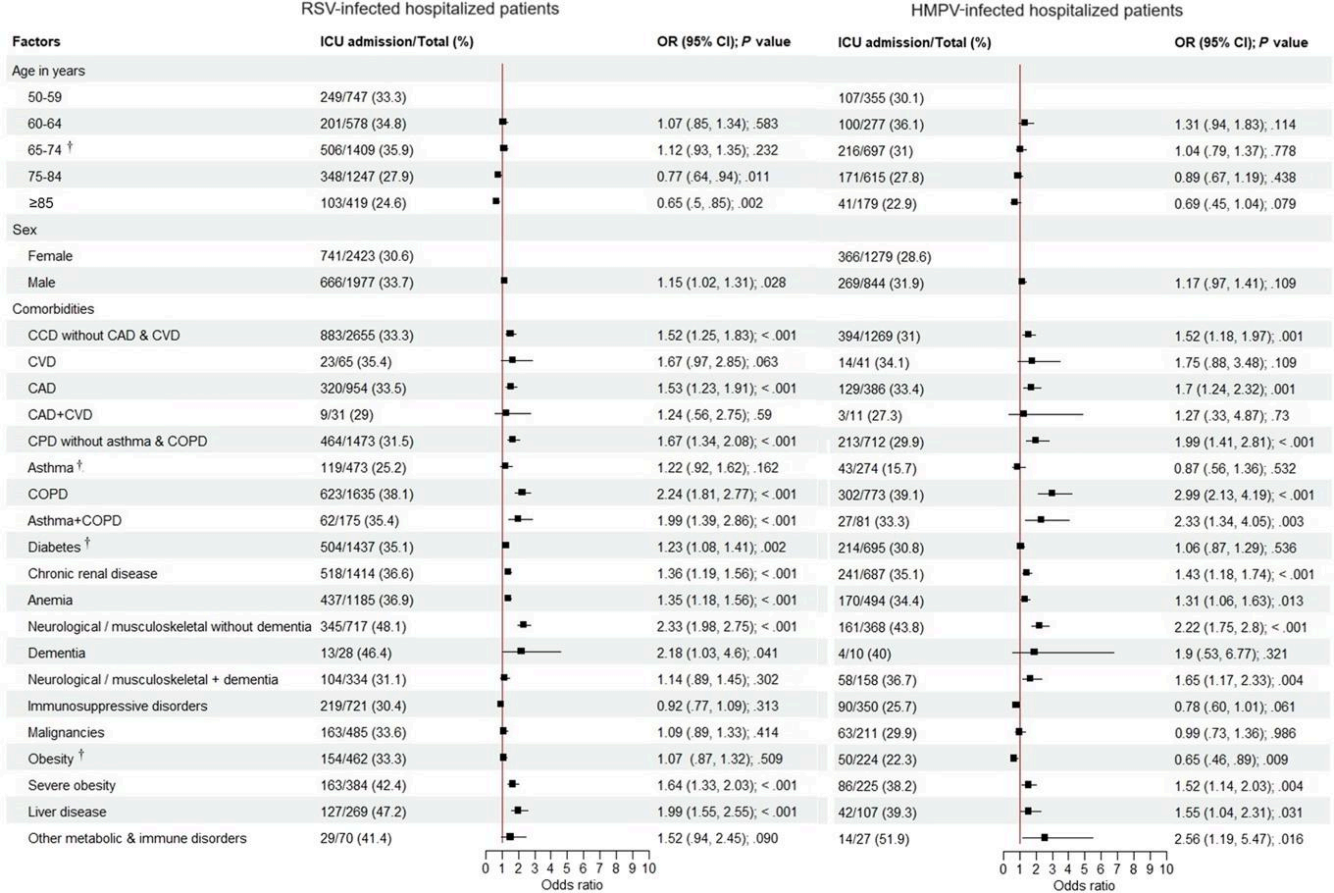
Forest plots showing the odds of intensive care unit admission associated with demographic parameters and comorbid conditions for patients hospitalized with respiratory syncytial virus or human metapneumovirus infection (2017–2023). ^†^Difference in proportions of ICU admissions between RSV and HMPV groups (*P* < .05). Abbreviations: CAD, coronary artery disease; CCD, chronic cardiac disease; CI, confidence interval; COPD, chronic pulmonary obstructive disease; CPD, chronic pulmonary disease; CVD, cerebrovascular disease; HMPV, human metapneumovirus; ICU, intensive care unit; OR, odds ratio; RSV, respiratory syncytial virus.

Comorbidity was assessed in reference to those with infection present without the given comorbidity present. The odds of ICU admission associated with demographic parameters and comorbid conditions for patients hospitalized with RSV or HMPV was evaluated as shown in Figure 2. The proportion of ICU admissions among patients hospitalized with RSV (1407/4400 [32%]) was similar to that for HMPV (635/2123 [29.9%]) (*P* = .09). In the RSV-infected group, patients aged ≥75 years showed a significantly reduced likelihood of ICU admission compared to the youngest group (adjusted odds ratio [aOR], 0.65 [95% CI, .50–.85]; *P* = .002). Males had significantly increased risk (aOR, 1.15 [95% CI, 1.02–1.31]). Patients with neurological/musculoskeletal disorders without dementia had the highest risk of ICU admission relative to those without these conditions (aOR, 2.33 [95% CI, 1.98–2.75]; *P* < .001), followed by patients with COPD (aOR, 2.24 [95% CI, 1.81–2.77]; *P* < .001) and dementia (aOR, 2.18 [95% CI, 1.03–4.60]; *P* < .001), compared to those without each respective comorbidity. Patients with asthma and COPD (aOR, 1.99 [95% CI, 1.39–2.86]; *P* < .001) and those with liver disease (aOR, 1.99 [95% CI, 1.55–2.55]; *P* < .001) had a similar magnitude of risk.

In contrast to the RSV group, in the HMPV group, the risk of ICU admission was not age or sex dependent. Contrasting with RSV, the highest risk was associated with COPD (aOR, 2.99 [95% CI, 2.13–4.19]; *P* < .001), followed by metabolic and immune disorders (aOR, 2.56 [95% CI, 1.19–5.47]; *P* = .016), asthma and COPD (aOR, 2.33 [95% CI, 1.34–4.05]; *P* = .003), and neurological/musculoskeletal disorders without dementia (aOR, 2.22 [95% CI, 1.75–2.80]; *P* < .001).

### Risk Factors for Mortality Among Patients Hospitalized With RSV and HMPV Infections

**Figure 3.**
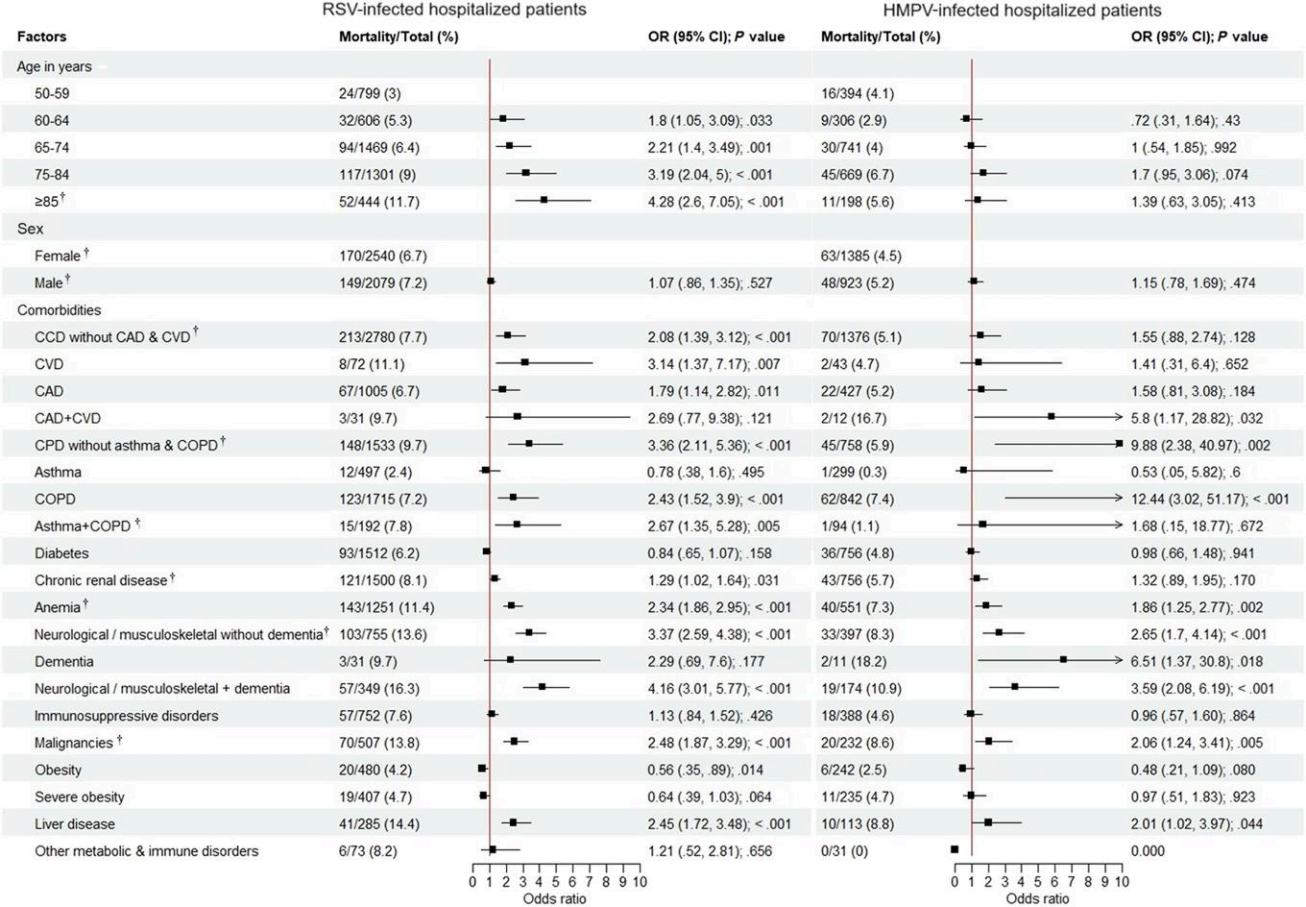
Forest plots showing the risk of mortality for hospitalized patients with respiratory syncytial virus or human metapneumovirus infection (2016–2023). ^†^Difference in the proportions of mortality between RSV and HMPV groups (*P* < .05). Abbreviations: CAD, coronary artery disease; CCD, chronic cardiac disease; CI, confidence interval; COPD, chronic pulmonary obstructive disease; CPD, chronic pulmonary disease; CVD, cerebrovascular disease; HMPV, human metapneumovirus; ICU, intensive care unit; OR, odds ratio; RSV, respiratory syncytial virus.

The risk of mortality for hospitalized patients infected with RSV or HMPV are shown in Figure 3. The proportion of in-hospital mortality was higher for those with RSV (319/4619 [6.9%]) than for HMPV (111/2308 [4.8%]) (*P* < .001). In those with RSV-associated hospitalizations, age, but not sex, was significantly associated with mortality, and patients with neurological/musculoskeletal disorders with dementia had the highest risk of dying (aOR, 4.16 [95% CI, 3.01–5.77]; *P* < .001). Patients with neurological/musculoskeletal disorder without dementia (aOR, 3.37 [95% CI, 2.59–4.38]; *P* < .001) and chronic pulmonary disease (CPD) without asthma and COPD (aOR, 3.36 [95% CI, 2.11–5.36]; *P* < .001) had a similar significant magnitude of risk. Other distinct risk groups for dying were those with cerebrovascular disease (CVD) (aOR, 3.14 [95% CI, 1.37–7.17]; *P* = .007), asthma and COPD (aOR, 2.67 [95% CI, 1.35–5.28]; *P* = .005), malignancies (aOR, 2.48 [95% CI, 1.87–3.29]; *P* < .001), and liver disease (aOR, 2.45 [95% CI, 1.72–3.48]; *P* < .001).

In the HMPV-infected group, age and sex had no effect on the risk of dying in the hospital. However, in contrast to RSV, COPD (aOR, 12.44 [95% CI, 3.02–51.17]; *P* < .001) had the highest risk, followed by CPD without asthma and COPD (aOR, 9.88 [95% CI, 2.38–40.97]; *P* = .002). Among cardiovascular factors, patients with both coronary artery disease and CVD (aOR, 5.80 [95% CI, 1.17–28.82]; *P* = .032) had a high risk of mortality. In the neurological category, patients with dementia alone (aOR, 6.51 [95% CI, 1.37–30.80]; *P* = .018), and neurological/musculoskeletal disorders with dementia (aOR, 3.59 [95% CI, 2.08–6.19]; *P* < .001) had elevated risks for dying, compared to HMPV-infected patients without these comorbidities.

### Risk of Outcomes by Number of Comorbid Conditions

**Figure 4.**
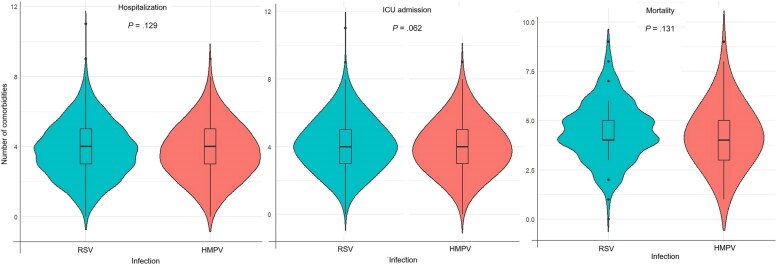
Distributions of the numbers of comorbidities for intensive care unit admission and mortality among hospitalized patients with respiratory syncytial virus or human metapneumovirus infection. Abbreviations: HMPV, human metapneumovirus; ICU, intensive care unit; RSV, respiratory syncytial virus.

The outcomes of ICU admission and mortality were also assessed according to the number of comorbidities of patients infected with RSV and HMPV. Overall, there was no difference in the distributions of the numbers of comorbidities for ICU admission and mortality when compared using violin plots (Figure 4). In the RSV group, the risks of both ICU admission and death increased significantly with increasing number of comorbidities as indicated by higher adjusted aOR for multiple comorbidities compared to the reference category of no comorbidities (Table 2).

**Table 2.** Odds of Outcomes According to Number of Comorbidities Among Hospitalized Patients With Respiratory Syncytial Virus– and Human Metapneumovirus–Associated Infections

Outcome	No. of Comorbidities	RSV Infection			HMPV Infection		
		No./Total (%)	OR (95% CI)	aOR (95% CI)^a^	No./Total (%)	OR (95% CI)	aOR (95% CI)^a^
ICU admission	≤1^††^	41/2099 (2.0)	Reference	Reference	24/511 (4.7)	Reference	Reference
	2–3^††^	455/2848 (16.0)	9.54 (6.89–13.21)**	9.87 (7.12–13.67)**	229/1183 (19.4)	4.87 (3.15–7.52)**	5.04 (3.26–7.79)**
	>3	916/2808 (32.6)	24.30 (17.66–33.43)**	25.16 (18.26–34.66)**	382/1291 (29.6)	8.53 (5.56–13.07)**	8.84 (5.76–13.66)**
Mortality	≤1	6/2120 (0.3)	Reference	Reference	4/527 (0.8)	Reference	Reference
	2–3	78/2929 (2.7)	9.64 (4.19–22.16)**	8.39 (3.65–19.32)**	30/1249 (2.4)	3.22 (1.13–9.18)*	2.95 (1.03–8.44)*
	>3^†^	246/2925 (8.4)	32.35 (14.36)**	27.76 (12.31–62.61)**	81/1397 (5.8)	8.05 (2.93–22.07)**	7.39 (2.69–20.34)**

Abbreviations: aOR, adjusted odds ratio; CI, confidence interval; HMPV, human metapneumovirus; ICU, intensive care unit; OR, odds ratio; RSV, respiratory syncytial virus.

^a^Obtained using multiple logistic regression model after adjusting for age and sex.

Difference in the proportions of outcome between RSV and HMPV groups: ^†^*P <* .05; ^††^*P* < .001.

*P < .05.

**P < .001.

In the HMPV-infected group also, the risk of ICU admission and mortality increased significantly with increasing numbers of comorbidities indicated by higher aOR associated with multiple comorbidities, as compared to the reference category of no comorbidities.

### Length of Hospital Stay

The median length of hospital stay for patients with either type of infection was 4 days with an interquartile range of 5 days and 4 days for the RSV and HMPV groups, respectively. In each infection group, the median length of stay increased significantly with the increasing number of comorbidities (*P* < .001; Supplementary Table 6), There were no differences in length of stay between RSV and HMPV groups when classified by comorbidity (Supplementary Table 7).

## DISCUSSION

In this large database study from Colorado, which spanned the COVID-19 pandemic, we examined 4619 RSV hospitalizations and almost half that number of HMPV hospitalizations. It is possible that the lower number of HMPV cases was due to the potential lower number tested for HMPV, since the detection of HMPV would require use of a more expensive multiplex testing panel, rather than the cheaper tests for influenza–RSV or influenza–RSV–SARS-CoV-2 combination tests. We did find some differences in the age distributions of hospitalized patients. For RSV, there appeared to be increasing proportions of hospitalization in older age groups, whereas with HMPV, not only was there no differential in the age distribution, but the proportions also appear to be higher in younger adults compared to RSV. These age distributions for RSV hospitalizations have been described previously, but not as well for HMPV [36]. We found a female predominance of RSV [37, 38] and HMPV [20, 37] hospitalizations, similar to other studies.

The seasonality of both respiratory viruses shows that at least for RSV, there appears to be an increasing number of cases every year. We are not sure if this reflects more RSV testing (in the pandemic years and thereafter) or if it mirrors the surges in pediatric disease. The postpandemic RSV seasonality in our study correlates very well with the pediatric peaks (a midsummer peak in 2022 and high peak in 2023) [21, 22]. It is well known that older adults are often infected by young children and when grandchildren visit them. In Colorado in the prepandemic years, the seasonality of HMPV coincided with RSV. This is in contrast with the usual pattern seen in many other studies, which tended to show that HMPV season follows the RSV season [39–41], a pattern parenthetically seen only in the postpandemic years in Colorado.

In this study, surprisingly, patients aged ≥75 years appeared to comprise a significantly lower proportion of admissions to the ICU than in the youngest age group for RSV. This probably reflects the higher rates of mortality in older patients, some of whom might have been sent to hospice. Other studies that followed patients outside the hospital for 30 days or up to a year later [36] reflect these out-of-hospital deaths and emphasize the need for longer-term follow-up of these patients. For HMPV, in keeping with the lack of age differential with ICU admission, we did not see an age differential in mortality consistent with other studies. This could be explained by potential differences in the presentations of disease with RSV and HMPV (eg, there was more pneumonia in HMPV patients). That more men were admitted to the ICU in the RSV group, but no sex differential was seen in the HMPV patients, might prompt investigations into the putative mechanisms of more severe HMPV disease in the older adult.

Given that as adults age the number of comorbidities increases, we showed a trend in every age group for increased risk of ICU admissions and mortality with increasing numbers of comorbidities. Indeed, for RSV and HMPV, there were significant trends in every age group. In every age group, for RSV the point estimates for ≥3 comorbidities were between 25 and 44 times higher than in those with no comorbidities or 1 comorbidity. For HMPV the differential was much less, at 7- to 16-fold higher (Table 3). We also showed significantly longer lengths of stay with increasing comorbidities (Supplementary Table 7).

Some of these differences in the effect of comorbidities might have been due to our broader definition of ARI, rather than a narrower definition of LRTI. We chose to use a broader definition for our inclusion of subjects (ILI, LRTI, and other ARI) because it has been proposed that narrower definitions do not necessarily capture the true burden of disease [42]; additionally, the clinical features of RSV and HMPV in older adults are often less specific [43] than in children, where RSV causes more well-defined clinical syndromes [44].

There are several limitations of claims database studies. First, we could not define the clinical presentation of patients as one would do in a prospective study. Similarly, the reference groups are different for the RSV and HMPV groups, so findings may not be directly comparable between infections. However, the ability to query the large database, with complete in-hospital follow-up, was a strength of the study. The database will not capture comorbidities that were not recorded in the current admission (eg, a previous heart attack or previous repeated admissions for COPD or asthma, which might be important predisposing conditions to this admission, cannot be captured). However, we did show very clearly that an increasing number of comorbidities clearly increases the duration of hospitalization and the number of older adults hospitalized in every age group for both RSV and HMPV; furthermore, that the claims database captures most comorbidities that are mentioned in the medical record allowed us to answer other types of questions, which could not be answered in prospective studies unless they were very large. Finally, even though we captured those older adults who were discharged moribund to hospice care, we were unable to determine whether they actually died.

## CONCLUSIONS

RSV and HMPV have differential risk factors for severity among older adult patients, many of which are not well known, especially for HMPV-infected patients. Our findings suggest that increasing age and male sex are more closely associated risks for RSV severity as determined by ICU admission and mortality outcomes, but is not necessarily the case for HMPV patients. The highest-risk comorbidities for mortality among both infections were primarily cardiovascular and pulmonary conditions, but especially the overlap of asthma plus COPD, as well as cerebrovascular conditions among RSV-infected patients. The number of comorbidities also increased the risk of both outcomes for both RSV and HMPV and may warrant further investigation, particularly with the addition of longitudinal data and/or clinical presentations.

## Supplementary Material

jiaf266_Supplementary_Data

## Supplementary Data

Supplementary materials are available at *The Journal of Infectious Diseases* online (http://jid.oxfordjournals.org/). Supplementary materials consist of data provided by the author that are published to benefit the reader. The posted materials are not copyedited. The contents of all supplementary data are the sole responsibility of the authors. Questions or messages regarding errors should be addressed to the author.

## Notes

***Acknowledgments*.** We acknowledge with thanks the many fruitful discussions we had with Drs Ajoke Sobanjo-ter Meulen, Niranjan Knesa-thesan, and Chengbin Wang of Icosavax (now AstraZeneca) and with both editors of this supplement, Drs Kathy Edwards and Angela Branche, for the development and refinement of this manuscript.

***Financial support.*** Funding for this study was obtained from Icosavax, a member of the AstraZeneca Group.

***Supplement sponsorship.*** This article appears as part of the supplement “Human Metapneumovirus Disease Burden in Older Adults and Implications for Adult Vaccination Strategies,” sponsored by AstraZeneca.

## References

[1] Falsey AR, Cunningham CK, Barker WH, et al Respiratory syncytial virus and influenza A infections in the hospitalized elderly. J Infect Dis 1995; 172:389–94. 7622882 10.1093/infdis/172.2.389

[2] Falsey AR, Hennessey PA, Formica MA, Cox C, Walsh EE. Respiratory syncytial virus infection in elderly and high-risk adults. N Engl J Med 2005; 352:1749–59. 15858184 10.1056/NEJMoa043951

[3] McLaughlin JM, Khan F, Begier E, Swerdlow DL, Jodar L, Falsey AR. Rates of medically attended RSV among US adults: a systematic review and meta-analysis. Open Forum Infect Dis 2022; 9:ofac300. 35873302 10.1093/ofid/ofac300 PMC9301578

[4] Surie D, Yuengling KA, DeCuir J, et al Severity of respiratory syncytial virus vs COVID-19 and influenza among hospitalized US adults. JAMA Netw Open 2024; 7:e244954. 38573635 10.1001/jamanetworkopen.2024.4954 PMC11192181

[5] May F, Ginige S, Firman E, et al Estimating the incidence of COVID-19, influenza and respiratory syncytial virus infection in three regions of Queensland, Australia, winter 2022: findings from a novel longitudinal testing-based sentinel surveillance programme. BMJ Open 2024; 14:e081793. 10.1136/bmjopen-2023-081793 PMC11043701 38653507

[6] Martinón-Torres F, Gutierrez C, Cáceres A, Weber K, Torres A. How does the burden of respiratory syncytial virus compare to influenza in Spanish adults? Influenza Other Respir Viruses 2024; 18:e13341. 38923767 10.1111/irv.13341 PMC11194680

[7] Hönemann M, Maier M, Frille A, et al Respiratory syncytial virus in adult patients at a tertiary care hospital in Germany: clinical features and molecular epidemiology of the fusion protein in the severe respiratory season of 2022/2023. Viruses 2024; 16:943. 38932235 10.3390/v16060943 PMC11209376

[8] Foley DA, Minney-Smith CA, Tjea A, et al The changing detection rate of respiratory syncytial virus in adults in Western Australia between 2017 and 2023. Viruses 2024; 16:656. 38793538 10.3390/v16050656 PMC11125702

[9] Moyes J, Tempia S, Walaza S, et al The attributable fraction of respiratory syncytial virus among patients of different ages with influenza-like illness and severe acute respiratory illness in a high HIV prevalence setting, South Africa, 2012–2016. Int J Infect Dis 2023; 134:71–7. 37211271 10.1016/j.ijid.2023.05.009 PMC10675839

[10] Shinkai M, Ota S, Ishikawa N, et al Burden of respiratory syncytial virus, human metapneumovirus and influenza virus infections in Japanese adults in the Hospitalized Acute Respiratory Tract Infection study. Respir Investig 2024; 62:717–25. 10.1016/j.resinv.2024.05.015 38823191

[11] Prasad N, Newbern EC, Trenholme AA, et al The health and economic burden of respiratory syncytial virus associated hospitalizations in adults. PLoS One 2020; 15:e0234235. 32525898 10.1371/journal.pone.0234235 PMC7289360

[12] Britton A, Roper LE, Kotton CN, et al Use of respiratory syncytial virus vaccines in adults aged ≥60 years: updated recommendations of the Advisory Committee on Immunization Practices—United States, 2024. MMWR Morb Mortal Wkly Rep 2024; 73:696–702. 39146277 10.15585/mmwr.mm7332e1

[13] Walsh EE, Peterson DR, Falsey AR. Human metapneumovirus infections in adults: another piece of the puzzle. Arch Intern Med 2008; 168:2489–96. 19064834 10.1001/archinte.168.22.2489 PMC2783624

[14] van den Hoogen BG, de Jong JC, Groen J, et al A newly discovered human pneumovirus isolated from young children with respiratory tract disease. Nat Med 2001; 7:719–24. 11385510 10.1038/89098 PMC7095854

[15] Widmer K, Zhu Y, Williams JV, Griffin MR, Edwards KM, Talbot HK. Rates of hospitalizations for respiratory syncytial virus, human metapneumovirus, and influenza virus in older adults. J Infect Dis 2012; 206:56–62. 22529314 10.1093/infdis/jis309 PMC3415933

[16] Zhao H, Green H, Lackenby A, et al A new laboratory-based surveillance system (Respiratory DataMart System) for influenza and other respiratory viruses in England: results and experience from 2009 to 2012. Euro Surveill 2014; 19:20680. 24480060 10.2807/1560-7917.es2014.19.3.20680

[17] Zhang Y, Sakthivel SK, Bramley A, et al Serology enhances molecular diagnosis of respiratory virus infections other than influenza in children and adults hospitalized with community-acquired pneumonia. J Clin Microbiol 2017; 55:79–89. 27795341 10.1128/JCM.01701-16 PMC5228265

[18] Radin JM, Katz MA, Tempia S, et al Influenza surveillance in 15 countries in Africa, 2006–2010. J Infect Dis 2012; 206(Suppl 1):S14–21. 23169960 10.1093/infdis/jis606

[19] Gupta V, Dawood FS, Rai SK, et al Validity of clinical case definitions for influenza surveillance among hospitalized patients: results from a rural community in north India. Influenza Other Respir Viruses 2013; 7:321–9. 22804843 10.1111/j.1750-2659.2012.00401.x PMC5779832

[20] Aminisani N, Wood T, Jelley L, Wong C, Huang QS. The burden of HMPV and influenza associated hospitalizations in adults in New Zealand, 2012–2015. J Infect Dis 2024; 230:933–43. 38349230 10.1093/infdis/jiae064

[21] Suss RJ, Simões EAF. Respiratory syncytial virus hospital-based burden of disease in children younger than 5 years, 2015–2022. JAMA Network Open 2024; 7:e247125. 38635270 10.1001/jamanetworkopen.2024.7125 PMC12068875

[22] Suss RJ, Simões EAF. An evaluation of 2015–2019 United States respiratory syncytial virus hospitalizations as a framework to develop potential strategies for the prevention of the hospital burden among infants. EClinicalMedicine 2024; 75:102790. 39257959 10.1016/j.eclinm.2024.102790 PMC11385787

[23] Choudhuri JA, Ogden LG, Ruttenber AJ, Thomas DSK, Todd JK, Simoes EAF. Effect of altitude on hospitalizations for respiratory syncytial virus infection. Pediatrics 2006; 117:349–56. 16452353 10.1542/peds.2004-2795

[24] Zachariah P, Ruttenber M, Simoes EAF. Hospitalizations due to respiratory syncytial virus in children with congenital malformations. Pediatr Infect Dis J 2011; 30:442–5. 21127456 10.1097/INF.0b013e318201813b

[25] Piedra PA, Gaglani MJ, Riggs M, et al Live attenuated influenza vaccine, trivalent, is safe in healthy children 18 months to 4 years, 5 to 9 years, and 10 to 18 years of age in a community-based, nonrandomized, open-label trial. Pediatrics 2005; 116:e397–407. 16140685 10.1542/peds.2004-2258 PMC1361119

[26] Greenbaum AH, Chen J, Reed C, et al Hospitalizations for severe lower respiratory tract infections. Pediatrics 2014; 134:546–54. 25113302 10.1542/peds.2014-0244

[27] Öner D, Vernhes C, Balla-Jhagjhoorsingh S, et al Serum and mucosal antibody-mediated protection and identification of asymptomatic respiratory syncytial virus infection in community-dwelling older adults in Europe. Front Immunol 2024; 15:1448578. 39493753 10.3389/fimmu.2024.1448578 PMC11527605

[28] Alfano F, Bigoni T, Caggiano FP, Papi A. Respiratory syncytial virus infection in older adults: an update. Drugs Aging 2024; 41:487–505. 38713299 10.1007/s40266-024-01118-9 PMC11193699

[29] Falsey AR . Human metapneumovirus infection in adults. Pediatr Infect Dis J 2008; 27:S80–3. 18820584 10.1097/INF.0b013e3181684dac

[30] Falsey AR, Erdman D, Anderson LJ, Walsh EE. Human metapneumovirus infections in young and elderly adults. J Infect Dis 2003; 187:785–90. 12599052 10.1086/367901

[31] Colorado Department of Local Affairs. Understanding Colorado regions. **2023**. Available at: https://demography.dola.colorado.gov/.

[32] Amand C, Tong S, Kieffer A, Kyaw MH. Healthcare resource use and economic burden attributable to respiratory syncytial virus in the United States: a claims database analysis. BMC Health Serv Res 2018; 18:294. 29678177 10.1186/s12913-018-3066-1 PMC5910575

[33] Hoshino T, Uchiyama S, Wong LKS, et al Five-year prognosis after TIA or minor ischemic stroke in Asian and non-Asian populations. Neurology 2021; 96:e54–66. 33046613 10.1212/WNL.0000000000010995

[34] Wadhera RK, Secemsky EA, Xu J, Yeh RW, Song Y, Goldhaber SZ. Community socioeconomic status, acute cardiovascular hospitalizations, and mortality in Medicare, 2003 to 2019. Circ Cardiovasc Qual Outcomes 2024; 17:e010090. 38597091 10.1161/CIRCOUTCOMES.123.010090 PMC12853152

[35] Woodruff RC, Melgar M, Pham H, et al Acute cardiac events in hospitalized older adults with respiratory syncytial virus infection. JAMA Intern Med 2024; 184:602–11. 38619857 10.1001/jamainternmed.2024.0212 PMC11019447

[36] Tseng HF, Sy LS, Ackerson B, et al Severe morbidity and short- and mid- to long-term mortality in older adults hospitalized with respiratory syncytial virus infection. J Infect Dis 2020; 222:1298–310. 32591787 10.1093/infdis/jiaa361

[37] Widmer K, Griffin MR, Zhu Y, Williams JV, Talbot HK. Respiratory syncytial virus- and human metapneumovirus–associated emergency department and hospital burden in adults. Influenza Other Respir Viruses 2014; 8:347–52. 24512531 10.1111/irv.12234 PMC3984605

[38] McClure DL, Kieke BA, Sundaram ME, et al Seasonal incidence of medically attended respiratory syncytial virus infection in a community cohort of adults ≥50 years old. PLoS One 2014; 9:e102586. 25025344 10.1371/journal.pone.0102586 PMC4099308

[39] Haynes AK, Fowlkes AL, Schneider E, Mutuc JD, Armstrong GL, Gerber SI. Human metapneumovirus circulation in the United States, 2008 to 2014. Pediatrics 2016; 137:e20152927. 27244790 10.1542/peds.2015-2927

[40] Seo YB, Song JY, Choi MJ, et al Etiology and clinical outcomes of acute respiratory virus infection in hospitalized adults. Infect Chemother 2014; 46:67–76. 25024868 10.3947/ic.2014.46.2.67 PMC4091371

[41] Cattoir L, Vankeerberghen A, Boel A, Van Vaerenbergh K, De Beenhouwer H. Epidemiology of RSV and hMPV in Belgium: a 10-year follow-up. Acta Clin Belg 2019; 74:229–35. 30029583 10.1080/17843286.2018.1492509

[42] Li Y, Kulkarni D, Begier E, et al Adjusting for case under-ascertainment in estimating RSV hospitalisation burden of older adults in high-income countries: a systematic review and modelling study. Infect Dis Ther 2023; 12:1137–49. 36941483 10.1007/s40121-023-00792-3 PMC10027261

[43] Branche AR, Falsey AR. Respiratory syncytial virus infection in older adults: an under-recognized problem. Drugs Aging 2015; 32:261–9. 25851217 10.1007/s40266-015-0258-9

[44] Simoes EA . Respiratory syncytial virus infection. Lancet 1999; 354:847–52. 10485741 10.1016/S0140-6736(99)80040-3

