# Supplementary material for: Respiratory Syncytial Virus and Human Metapneumovirus Respiratory Hospitalizations and Outcomes in Colorado Adults ≥50 Years of Age: 2016–2023
Source: J Infect Dis. 2025 Jul 16;232(Suppl 1):S19–28. doi: 10.1093/infdis/jiaf266 (PMC12265064; doi:10.1093/infdis/jiaf266)
Supplement: jiaf266_Supplementary_Data [file jiaf266_supplementary_data.docx]

**Supplementary Table 1.** Annual RSV- and HMPV-associated Hospitalizations by Colorado Region, 2016-2023

|  | **Annual RSV Hospitalizations by Region**  **N (% of Annual Total)** | | | | | | | | | | |
| --- | --- | --- | --- | --- | --- | --- | --- | --- | --- | --- | --- |
| **Year** | 2016 | 2017 | 2018 | 2019 | 2020 | 2021 | 2022 | 2023 | Total | Total RSV+HMPV |  |
| **Region** |  |  |  |  |  |  |  |  |  |  |  |
| Eastern Plains | 2 (0.93) | 1 (0.21) | 12 (1.97) | 4 (0.53) | 4 (0.69) | 6 (1.32) | 14 (1.50) | 10 (1.68) | 53 (1.15) | 78 (1.13) |  |
| Front Range | 200 (92.59) | 432 (90.38) | 530 (87.03) | 668 (89.30) | 523 (89.71) | 400 (87.72) | 830 (89.06) | 511 (86.03) | 4,097 (88.70) | 6,136 (88.58) |  |
| Central Mountains | (0.0) | 1 (0.21) | 2 (0.33) | 5 (0.67) | 6 (1.03) | 1 (0.22) | 8 (0.86) | 10 (1.68) | 33 (0.71) | 37 (0.53) |  |
| San Luis Valley | (0.0) | 1 (0.21) | 13 (2.13) | 11 (1.47) | 9 (1.54) | 3 (0.66) | 8 (0.86) | 5 (0.84) | 50 (1.08) | 76 (1.10) |  |
| Western Slope | 14 (6.48) | 43 (9.0) | 52 (8.54) | 60 (8.02) | 41 (7.03) | 46 (10.09) | 72 (7.73) | 58 (9.76) | 386 (8.36) | 600 (8.66) |  |
| Annual total | 216 (100.0) | 478 (100.0) | 609 (100.0) | 748 (100.0) | 583 (100.0) | 456 (100.0) | 932 (100.0) | 594 (100.0) | 4,619 (100.0) | 6,927 (100.0) |  |
|  | **Annual HMPV Hospitalizations by Region N (% of Annual Total)** | | | | | | | | | |  |
| **Year** | 2016 | 2017 | 2018 | 2019 | 2020 | 2021 | 2022 | 2023 | Total | Total RSV+HMPV |  |
| **Region** |  |  |  |  |  |  |  |  |  |  |  |
| Eastern Plains | 0 (0.0) | 0 (0.0) | 2 (0.50) | 2 (0.45) | 10 (2.79) | 0 (0.0) | 2 (0.71) | 9 (1.94) | 25 (1.08) | 78 (1.13) |  |
| Front Range | 163 (89.07) | 115 (79.31) | 374 (93.03) | 411 (92.99) | 310 (86.35) | 25 (80.65) | 233 (83.21) | 406 (87.50) | 2,039 (88.34) | 6,136 (88.58) |  |
| Central Mountains | 0 (0.0) | 0 (0.0) | 0 (0.0) | 0 (0.0) | 3 (0.84) | 0 (0.0) | 1 (0.36) | 0 (0.0) | 4 (0.17) | 37 (0.53) |  |
| San Luis Valley | 0 (0.0) | 8 (5.52) | 3 (0.75) | 2 (0.45) | 4 (1.11) | 0 (0.0) | 5 (1.79) | 4 (0.86) | 26 (1.13) | 76 (1.10) |  |
| Western Slope | 20 (10.93) | 22 (15.17) | 23 (5.72) | 27 (6.11) | 32 (8.91) | 6 (19.35) | 39 (13.93) | 45 (9.70) | 214 (9.27) | 600 (8.66) |  |
| Annual total | 183 (84.72) | 145 (30.33) | 402 (66.01) | 442 (59.09) | 359 (61.58) | 31 (6.80) | 280 (30.04) | 464 (78.11) | 2,308 (49.97) | 6,927 (100.0) |  |

**Supplementary Table 2:** Distribution of patients **with RSV** and **HMPV** associated hospitalizations by **age** and **year of admission** [n (%)]

| Infection type | Age categories | 2016 | 2017 | 2018 | 2019 | 2020 | 2021 | 2022 | 2023 |
| --- | --- | --- | --- | --- | --- | --- | --- | --- | --- |
| RSV | 50-59 | 57(24.6) | 104(18.6) | 158(20.3) | 194(19.2) | 148(17.2) | 231(22.1) | 421(20.1) | 282(20.4) |
|  | 60-64 | 27(11.6) | 85(15.2) | 94(12.1) | 132(13.1) | 125(14.6) | 135(12.9) | 312(14.9) | 194(14) |
|  | 65-74 | 66(28.4) | 170(30.4) | 238(30.6) | 294(29.1) | 292(34) | 331(31.6) | 688(32.8) | 425(30.8) |
|  | 75-84 | 56(24.1) | 141(25.2) | 220(28.2) | 290(28.7) | 223(26) | 278(26.6) | 540(25.8) | 376(27.2) |
|  | >= 85 | 26(11.2) | 59(10.6) | 69(8.9) | 100(9.9) | 71(8.3) | 71(6.8) | 136(6.5) | 104(7.5) |
|  | Total | 232(100) | 559(100) | 779(100) | 1010(100) | 859(100) | 1046(100) | 2097(100) | 1381(100) |
| HMPV | 50-59 | 37(19.8) | 38(20.9) | 83(17.6) | 101(18.1) | 87(17.5) | 12(22.2) | 89(19.3) | 123(16.1) |
|  | 60-64 | 29(15.5) | 27(14.8) | 53(11.3) | 65(11.6) | 75(15.1) | 5(9.3) | 73(15.9) | 98(12.9) |
|  | 65-74 | 48(25.7) | 54(29.7) | 149(31.6) | 183(32.7) | 158(31.9) | 21(38.9) | 148(32.2) | 248(32.5) |
|  | 75-84 | 54(28.9) | 47(25.8) | 140(29.7) | 156(27.9) | 145(29.2) | 11(20.4) | 112(24.3) | 231(30.3) |
|  | >= 85 | 19(10.2) | 16(8.8) | 46(9.8) | 54(9.7) | 31(6.3) | 5(9.3) | 38(8.3) | 62(8.1) |
|  | Total | 187(100) | 182(100) | 471(100) | 559(100) | 496(100) | 54(100) | 460(100) | 762(100) |

*Percentage is according to number of infected patients in each age group per year*

**Supplementary Table 3:** Proportion of patients with RSV associated hospitalization with comorbidities by year of admission [n (%)]

| Comorbidities | Epiyear | | | | | | | |
| --- | --- | --- | --- | --- | --- | --- | --- | --- |
|  | 2016 | 2017 | 2018 | 2019 | 2020 | 2021 | 2022 | 2023 |
| CCD with no CVD and no CAD | 128(55.2) | 348(62.3) | 467(59.9) | 545(54) | 484(56.3) | 535(51.1) | 1003(47.8) | 704(51) |
| Cerebrovascular disease | 7(3) | 18(3.2) | 8(1) | 26(2.6) | 12(1.4) | 4(0.4) | 27(1.3) | 13(0.9) |
| CAD | 51(22) | 114(20.4) | 149(19.1) | 201(19.9) | 170(19.8) | 152(14.5) | 292(13.9) | 181(13.1) |
| CPD with no Asthma and no COPD | 61(26.3) | 123(22) | 196(25.2) | 260(25.7) | 234(27.2) | 223(21.3) | 508(24.2) | 330(23.9) |
| Asthma and no COPD | 43(18.5) | 120(21.5) | 111(14.2) | 139(13.8) | 106(12.3) | 136(13) | 228(10.9) | 170(12.3) |
| COPD with no Asthma | 99(42.7) | 264(47.2) | 330(42.4) | 390(38.6) | 319(37.1) | 256(24.5) | 488(23.3) | 348(25.2) |
| Both Asthma and COPD | 18(7.8) | 52(9.3) | 28(3.6) | 28(2.8) | 21(2.4) | 18(1.7) | 34(1.6) | 17(1.2) |
| Diabetes | 78(33.6) | 183(32.7) | 249(32) | 288(28.5) | 251(29.2) | 287(27.4) | 547(26.1) | 303(21.9) |
| CRD | 87(37.5) | 194(34.7) | 242(31.1) | 330(32.7) | 266(31) | 203(19.4) | 402(19.2) | 230(16.7) |
| Anemia | 66(28.4) | 143(25.6) | 169(21.7) | 209(20.7) | 170(19.8) | 150(14.3) | 292(13.9) | 178(12.9) |
| Neurological disorder and no Dementia | 38(16.4) | 77(13.8) | 92(11.8) | 138(13.7) | 110(12.8) | 87(8.3) | 202(9.6) | 120(8.7) |
| Dementia | 18(7.8) | 50(8.9) | 48(6.2) | 83(8.2) | 55(6.4) | 44(4.2) | 93(4.4) | 64(4.6) |
| Neurological and Dementia | 15(6.5) | 48(8.6) | 46(5.9) | 76(7.5) | 51(5.9) | 40(3.8) | 88(4.2) | 58(4.2) |
| Immunosuppressive disorder | 32(13.8) | 90(16.1) | 120(15.4) | 145(14.4) | 100(11.6) | 104(9.9) | 193(9.2) | 127(9.2) |
| Malignancies | 22(9.5) | 59(10.6) | 65(8.3) | 104(10.3) | 72(8.4) | 64(6.1) | 155(7.4) | 83(6) |
| Obesity | 18(7.8) | 46(8.2) | 66(8.5) | 91(9) | 75(8.7) | 66(6.3) | 143(6.8) | 94(6.8) |
| Severe obesity | 24(10.3) | 46(8.2) | 42(5.4) | 55(5.4) | 59(6.9) | 50(4.8) | 105(5) | 80(5.8) |
| Liver | 15(6.5) | 21(3.8) | 34(4.4) | 52(5.1) | 39(4.5) | 45(4.3) | 75(3.6) | 48(3.5) |
| Other metabolic & immune disorders | 2(0.9) | 7(1.3) | 11(1.4) | 14(1.4) | 7(0.8) | 8(0.8) | 23(1.1) | 8(0.6) |

**Supplementary Table 4:** Proportion of patients with HMPV associated hospitalizations by year of admission [n (%)]

| Comorbidities | Epiyear | | | | | | | |
| --- | --- | --- | --- | --- | --- | --- | --- | --- |
|  | 2016 | 2017 | 2018 | 2019 | 2020 | 2021 | 2022 | 2023 |
| CCD with no CVD and no CAD | 107(57.2) | 105(57.7) | 271(57.5) | 329(58.9) | 291(58.7) | 28(51.9) | 245(53.3) | 408(53.5) |
| Cerebrovascular disease | 3(1.6) | 3(1.6) | 22(4.7) | 8(1.4) | 7(1.4) | 1(1.9) | 8(1.7) | 13(1.7) |
| CAD | 42(22.5) | 34(18.7) | 88(18.7) | 112(20) | 61(12.3) | 11(20.4) | 60(13) | 137(18) |
| CPD with no Asthma and no COPD | 46(24.6) | 46(25.3) | 133(28.2) | 166(29.7) | 146(29.4) | 19(35.2) | 156(33.9) | 216(28.3) |
| Asthma and no COPD | 39(20.9) | 32(17.6) | 78(16.6) | 96(17.2) | 58(11.7) | 4(7.4) | 68(14.8) | 128(16.8) |
| COPD with no Asthma | 83(44.4) | 65(35.7) | 202(42.9) | 220(39.4) | 170(34.3) | 14(25.9) | 116(25.2) | 237(31.1) |
| Both Asthma and COPD | 14(7.5) | 7(3.8) | 20(4.2) | 16(2.9) | 14(2.8) | 2(3.7) | 10(2.2) | 25(3.3) |
| Diabetes | 62(33.2) | 58(31.9) | 142(30.1) | 173(30.9) | 147(29.6) | 12(22.2) | 116(25.2) | 240(31.5) |
| CRD | 70(37.4) | 66(36.3) | 174(36.9) | 174(31.1) | 138(27.8) | 10(18.5) | 100(21.7) | 170(22.3) |
| Anemia | 56(29.9) | 40(22) | 108(22.9) | 96(17.2) | 86(17.3) | 14(25.9) | 73(15.9) | 133(17.5) |
| Neurological disorder and no Dementia | 29(15.5) | 22(12.1) | 86(18.3) | 76(13.6) | 68(13.7) | 5(9.3) | 52(11.3) | 102(13.4) |
| Dementia | 17(9.1) | 14(7.7) | 38(8.1) | 44(7.9) | 31(6.3) | 2(3.7) | 22(4.8) | 55(7.2) |
| Neurological and Dementia | 16(8.6) | 13(7.1) | 37(7.9) | 42(7.5) | 29(5.8) | 2(3.7) | 22(4.8) | 49(6.4) |
| Immunosuppressive disorder | 38(20.3) | 20(11) | 73(15.5) | 93(16.6) | 54(10.9) | 11(20.4) | 71(15.4) | 102(13.4) |
| Malignancies | 21(11.2) | 11(6) | 40(8.5) | 55(9.8) | 45(9.1) | 2(3.7) | 39(8.5) | 55(7.2) |
| Obesity | 18(9.6) | 25(13.7) | 38(8.1) | 50(8.9) | 41(8.3) | 8(14.8) | 44(9.6) | 70(9.2) |
| Severe obesity | 9(4.8) | 20(11) | 40(8.5) | 56(10) | 29(5.8) | 1(1.9) | 30(6.5) | 67(8.8) |
| Liver | 6(3.2) | 5(2.7) | 22(4.7) | 23(4.1) | 17(3.4) | 3(5.6) | 18(3.9) | 40(5.2) |
| Other metabolic & immune disorders | 3(1.6) | 2(1.1) | 2(0.4) | 5(0.9) | 5(1) | 0(0) | 7(1.5) | 11(1.4) |

| **Supplementary Table 5:** Proportion of RSV and HMPV associated Hospital encpounters with Influenza infections, 2016-2023, N/Total (%) | | | |
| --- | --- | --- | --- |
|  |  |  |  |
|  | **RSV+Influenza** | **HMPV+Influenza** | **Total** |
| **Age (Years)** | **Hospitalization** | | |
| 50-59 | 22/777 (2.83) | 9/385 (2.34) | 31/1,162 (2.67) |
| 60-64 | 8/598 (1.34) | 2/304 (0.66) | 10/902 (1.11) |
| 65-74 | 31/1,438 (2.16) | 13/728 (1.79) | 44/2,166 (2.03) |
| 75-84 | 24/1,277 (1.88) | 7/662 (1.06) | 31/1,939 (1.60) |
| 85+ | 5/439 (1.14) | /198 (0.0) | 5/637 (0.78) |
| Total | 90/4,529 (1.99) | 31/2,277 (1.36) | 121/6,806 (1.78) |
| **Age (Years)** | **ICU Admission** | | |
| 50-59 | 7/242 (2.89) | 4/103 (3.88) | 11/345 (3.19) |
| 60-64 | 4/197 (2.03) | 1/99 (1.01) | 5/296 (1.69) |
| 65-74 | 15/493 (3.04) | 1/215 (0.47) | 16/708 (2.26) |
| 75-84 | 5/345 (1.45) | 2/169 (1.18) | 7/514 (1.36) |
| 85+ | 2/102 (1.96) | 0/41 (0.0) | 2/143 (1.40) |
| Total | 33/1,379 (2.39) | 8/627 (1.28) | 41/2,006 (2.04) |
| **Age (Years)** | **Death** | | |
| 50-59 | 2/23 (8.70) | None | 2/39 (5.13) |
| 60-64 | 2/33 (6.06) |  | 2/42 (4.76) |
| 65-74 | 2/96 (2.08) |  | 2/127 (1.57) |
| 75-84 | 1/116 (0.86) |  | 1/164 (0.61) |
| 85+ | 7/55 (12.73) |  | 7/66 (10.61) |
| Total | 14/323 (4.33) | 0/115 (0.0) | 14/438 (3.20) |

**Supplementary Table 6.** Comparison of length of stay between RSV and HMPPV associated hospitalization

based on the number of comorbidities

| Number of comorbidities | Respiratory Syncytial Virus [N=4619] | | | Human metapneumo virus [N=2308] | | | P-value^a^ |
| --- | --- | --- | --- | --- | --- | --- | --- |
|  | n | Median | IQR | n | Median | IQR |  |
| 0 | 32 | 2.00 | [1.00, 3.00] | 25 | 2.00 | [1.00, 2.00] | 0.191 |
| 1 | 259 | 3.00 | [2.00, 4.00] | 144 | 3.00 | [2.00, 4.50] | 0.496 |
| 2-3 | 1764 | 4.00 | [2.00, 6.00] | 904 | 3.50 | [2.00, 6.00] | 0.566 |
| > 3 | 2564 | 4.00 | [3.00, 8.00] | 1235 | 4.00 | [3.00, 7.00] | 0.679 |
| P-value^b^ |  | **< 0.001** |  |  | **< 0.001** |  |  |

^a^Obtained using Mann-Whitney U test; ^b^Obtained using Kruskal-Wallis test.

**Supplementary Table 7.** Descriptive statistics for length of hospital stay for RSV associated hospitalized patients by comorbidity

| Comorbidity^a^ | RSV - Length of stay (days) | | | | | HMPV - Length of stay (days) | | | | P-value^b^ |
| --- | --- | --- | --- | --- | --- | --- | --- | --- | --- | --- |
|  | n | Median | | 95% CI (Median) | | n | Median | 95% CI (Median) | |  |
| CCD and no CVD no CAD | 2780 | 4.00 | 4.00 | | 5.00 | 1376 | 4.00 | 4.00 | 5.00 | 0.694 |
| CVD | 72 | 5.00 | 5.00 | | 7.00 | 43 | 5.00 | 4.00 | 7.00 | 0.812 |
| CAD | 1005 | 4.00 | 4.00 | | 5.00 | 427 | 4.00 | 4.00 | 5.00 | 0.991 |
| CVD and CAD | 31 | 5.00 | 5.00 | | 9.00 | 12 | 5.50 | 3.00 | 11.00 | 0.744 |
| CPD and no Asthma no COPD | 1533 | 4.00 | 4.00 | | 5.00 | 758 | 4.00 | 4.00 | 5.00 | 0.107 |
| Asthma | 497 | 3.00 | 3.00 | | 4.00 | 299 | 3.00 | 3.00 | 4.00 | 0.087 |
| COPD | 1715 | 4.00 | 4.00 | | 5.00 | 842 | 5.00 | 5.00 | 6.00 | 0.119 |
| Asthma and with COPD | 192 | 5.00 | 5.00 | | 6.00 | 94 | 5.00 | 5.00 | 7.00 | 0.902 |
| Diabetes | 1512 | 4.00 | 4.00 | | 5.00 | 756 | 4.00 | 4.00 | 5.00 | 0.198 |
| CRD | 1500 | 4.00 | 4.00 | | 5.00 | 756 | 4.00 | 4.00 | 5.00 | 0.280 |
| Anemia | 1251 | 5.00 | 5.00 | | 6.00 | 551 | 5.00 | 5.00 | 6.00 | 0.752 |
| Neurological/Musculoskeletal but no dementia | 755 | 5.00 | 5.00 | | 6.00 | 397 | 5.00 | 5.00 | 6.00 | 0.725 |
| Dementia | 31 | 6.00 | 5.00 | | 9.00 | 11 | 5.00 | 3.00 | 8.00 | 0.584 |
| Neurological/Musculoskeletal and dementia | 349 | 5.00 | 5.00 | | 6.00 | 174 | 5.00 | 5.00 | 6.00 | 0.891 |
| Immunogenicity | 752 | 4.00 | 4.00 | | 5.00 | 388 | 4.00 | 4.00 | 5.00 | 0.279 |
| Malignancies | 507 | 4.00 | 4.00 | | 5.00 | 232 | 4.00 | 4.00 | 5.00 | 0.327 |
| Obesity | 480 | 4.00 | 4.00 | | 5.00 | 242 | 4.00 | 4.00 | 5.00 | 0.862 |
| Severe obesity | 407 | 5.00 | 5.00 | | 6.00 | 235 | 5.00 | 5.00 | 6.00 | 0.402 |
| Liver | 285 | 5.00 | 5.00 | | 6.00 | 113 | 5.00 | 5.00 | 7.00 | 0.285 |
| Other metabolic & immune disorders | 73 | 5.00 | 4.00 | | 7.00 | 31 | 5.00 | 3.00 | 7.00 | 0.872 |

^a^Comorbid conditions; CCD: Chronic cardiac disease; CAD: Coronary artery disease; CVD: Cerebrovascular disease; CPD: Chronic pulmonary disease; COPD: Chronic pulmonary obstructive disease; ^b^Obtained using Mann-Whitney U test.
